# Association between *EPAS1* and *ATP6V1E2* polymorphisms and susceptibility to high altitude polycythemia in Chinese Tibetan population

**DOI:** 10.3389/fmed.2025.1737704

**Published:** 2026-01-20

**Authors:** Lirong Ran, Yongjie Li, Dongwei Liao, Ziyi Chen, Guangming Wang, Yuanyuan Zhang

**Affiliations:** 1 School of Clinical Medicine, Dali University, Dali, Yunnan, China; 2 The First Affiliated Hospital of Dali University, Dali, Yunnan, China

**Keywords:** ATP6V1E2, EPAS1, genetic susceptibility, high altitude polycythemia, Tibetan

## Abstract

High altitude polycythemia (HAPC) is an important public health problem at high altitude, and genetic factors play a key role in hypoxia adaptation in Tibetan populations. The aim of this study was to investigate the association between *EPAS1* and *ATP6V1E2* gene locus polymorphisms and genetic susceptibility to HAPC in Chinese Tibetan population. This study included 78 HAPC patients and 85 healthy controls and genotyped the *EPAS1* gene single nucleotide polymorphism loci (rs1868092, rs4953396, and rs4953354) and *ATP6V1E2* rs896210. We analysed the association between *EPAS1* and *ATP6V1E2* genes and HAPC using logistic regression analysis Multifactorial dimensionality reduction, protein–protein interaction and KEGG pathway. This study found that *ATP6V1E2* rs896210 and *EPAS1* rs1868092, rs4953396, rs4953354 were significantly associated with genetic susceptibility to HAPC in the Chinese Tibetan population, and synergistic effects existed among these genetic loci. This provides new evidence for the genetic mechanism of high altitude adapted diseases in Tibetan populations, which is valuable for individualized risk assessment and exploration of potential therapeutic targets for HAPC at high altitude.

## 1 Introduction

Globally, more than 140 million people live at altitudes above 2,500 m, mainly in the Tibetan Plateau, the Andes and the Ethiopian Plateau (1). When humans live at high altitude, they may develop high altitude polycythemia (HAPC) due to overcompensated proliferation of red blood cells caused by hypoxia, which is one of the typical chronic alpine diseases and has been a serious public problem at high altitudes (2). HAPC leads to a significant increase in blood viscosity, which results in microcirculatory damage and immune response disturbances such as vascular thrombosis, widespread organ damage and sleep disorders (3, 4). Central to the development of HAPC is the over compensatory proliferation of erythrocytes, and erythropoiesis is largely dependent on erythropoietin (EPO), a glycoprotein hormone regulated by hypoxia-inducible factor (HIF) (5). It is noteworthy that the risk of developing HAPC varies significantly among different indigenous high-altitude populations. For instance, compared to residents of the South American Andes, the indigenous Tibetan population of the Qinghai-Tibet Plateau exhibits superior hypoxic adaptation, with a relatively lower incidence of HAPC (6–8). This disparity suggests that different populations may have evolved distinct genetic adaptation mechanisms to high-altitude environments. It has been shown that *EPAS1*, *ATP6V1E2* and other genes regulate the HIF pathway, and mutations in these loci have been shown to correlate with genetic susceptibility to HAPC in Tibetan populations (9–11).

Yi X et al. found population-specific variations in allele frequencies of several genes by whole exome sequencing of 50 Tibetan individuals, with the endothelial PAS structural domain protein 1 (*EPAS1*) gene showing the strongest signal of natural selection (12). The gene is located on chromosome 2p16-21 and is expressed mainly in tissues and organs involved in metabolism and oxygen supply, such as the placenta, vascular endothelium, and kidney (13–15). Previous studies have demonstrated that multiple single nucleotide polymorphisms (SNPs) in the EPAS1 gene—including rs1868092, rs4953396, and rs4953354—are significantly associated with hypoxic adaptation in the Tibetan population. However, the mechanisms underlying their association with high-altitude hypoxic anemia (HAPC) require further elucidation (16, 17).

*ATP6V1E2* (also known as *ATP6E1* or *VMA4*) is located on chromosome 2p21,and encodes the E subunit of the V-ATPase complex, which is a proton pump that regulates intracellular acid–base homeostasis (18). There are fewer direct studies on *ATP6V1E2* and hypoxia, but several lines of evidence suggest that this gene may be involved in high altitude hypoxia acclimatization. Whole-exome sequencing of the Tibetan population revealed significant racial differences in allele frequencies of *ATP6V1E2* (12),and significant correlations between the *ATP6V1E2* locus and levels of red blood cell counts (RBC), hemoglobin (HGB), and hematocrit (HTC), which are important features of hypoxic acclimatization in high altitude populations (9). Notably, *ATP6V1E2* is located in close proximity to the *EPAS1* gene, which is a known key gene for high altitude acclimatization, suggesting a possible synergistic regulatory relationship.

Genetic studies of Tibetans at high altitude in China have shown that the *EPAS1* and *ATP6V1E2* genes exhibit significant adaptive evolution under the selective pressure of prolonged high altitude. Although existing studies have confirmed the association of polymorphisms in these two genes with the risk of HAPC in Tibetan populations, the relevant loci are still understudied. In this study, we analyzed the genetic polymorphisms of *EPAS1* rs1868092, rs4953396, rs4953354 and *ATP6V1E2* rs896210 in the Chinese Tibetan population and their associations with the susceptibility to HAPC, which will provide more theoretical basis for early screening and individualized prevention and treatment of HAPC in the Tibetan population in this region.

## 2 Materials and methods

### 2.1 Study subjects

**Figure 1:**
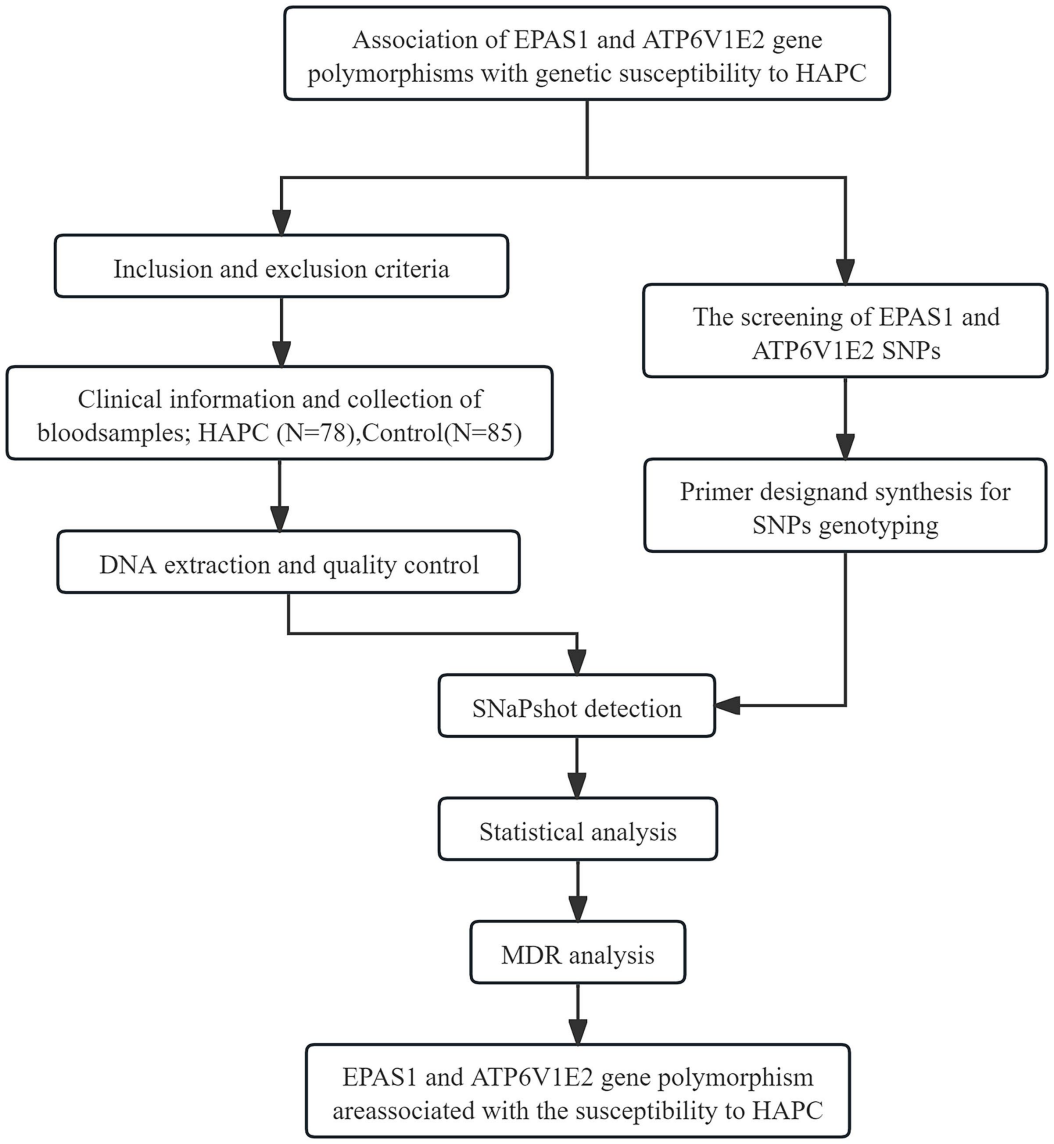
Flow chart of this study.

A total of 163 subjects who visited Tibet Autonomous Region People’s Hospital participated in this study. Samples data were accessed between 01/01/2018 and 31/12/2022 for the purpose of our study. Authors who were involved in recruitment, screening and conducting the experiments had access to information that could identify individual participants during data collection. This study included 78 patients with HAPC in the case group and 85 healthy individuals in the control group. The criteria for inclusion in the case group were (19): (1) Hb ≥ 21 g/dl for men and ≥ 19 g/dl for women; (2) Long-term residence in a high altitude area at an altitude of more than 3,000 m; (3) Three or more of the following symptoms: headache, dizziness, fatigue, cyanosis, sleep disturbance, conjunctival congestion, and purplish skin; (4) The study population excludes true cytokinesis as well as other secondary erythropoiesis. None of the study subjects had cardiovascular diseases, autoimmune diseases, malignant tumors, immune system diseases, or neurological diseases. Clinical data related to the two groups were also collected: gender, age, RBC, HGB, and HCT. All subjects are the Tibetan population in Lhasa, Tibet, China. The study was conducted in accordance with the principles of the Declaration of Helsinki and approved by the Research Ethics Committee of the First Affiliated Hospital of Dali University (Approval No. DFY20171210002, Date: December 10, 2017) and all participants provided written informed consent. The flowchart of this study is shown in Figure 1.

### 2.2 Experimental methods

#### 2.2.1 Reagents and instruments

DNA extraction kit (QIAGEN, lot 166,034,547); PCR primers (Life); Taq enzyme (Fermentas); qPCR premix (General Biologicals, lot FP208); PCR 96-well plate (Axygen, PCR-96-FLT-C); real-time PCR (Axygen, lot 166,034,547); and a PCR plate (Axygen, lot 166,034,547). PCR (BioRad, model: CFX96); ND5000 ultra-micro spectrophotometer (Thermo, model: NanoDrop 5,000); electrophoresis instrument (Major Science, model: Mini Pro 300 V Power Supply).

#### 2.2.2 Selection of SNP

The screening of SNPs in this study was based on reference information from the National Center for Biotechnology Information (NCBI, https://www.ncbi.nlm.nih.gov/) database. We focused on the following loci selected for analysis: *ATP6V1E2* rs896210 (*A/G*) and *EPAS1* rs1868092 (*A/G*), rs4953396 (*A/C*), rs4953354 (*A/G)*. It should be noted in particular that although the gene annotation information for the rs4953396 locus in the NCBI database is incomplete, it has been confirmed that this locus is located in the *EPAS1* gene region (20, 21), and therefore we included it in the locus analysis of the *EPAS1* gene.

#### 2.2.3 DNA extraction and quality control

Collect 2 mL of fasting venous blood from subjects into EDTA anticoagulant tubes and store at −20 °C for later use. Extract genomic DNA using a DNA extraction kit, then assess integrity via agarose gel electrophoresis and determine concentration and purity using a micro-spectrophotometer. Genotyping samples must meet quality control standards: concentration ≥20 ng/μL, A₂₆₀/A₂₈₀ ratio 1.8–2.0. The measured DNA concentrations in this study ranged from 0.9 to 1171.0 ng/μL, with A₂₆₀/A₂₈₀ ratios between 0.68 and 2.36. All samples ultimately included met the criteria, with ratios concentrated between 1.7 and 1.9. A few samples initially deviating from the criteria were included after purification and retesting.

#### 2.2.4 SNP detection

PCR amplification was first performed and the primer probe sequences for *EPAS1* rs1868092, rs4953396, rs4953354 and *ATP6V1E2* rs896210 are shown in Table 1. The reaction conditions were as follows: pre-denaturation at 95 °C for 5 min, 40 cycles (denaturation at 95 °C for 10 s, annealing at 60 °C for 30 s, extension at 72 °C for 2 min), and finally extension at 16 °C for 5 min. Then TaqMan fluorescent probe technology was used for genotyping polymorphic loci, and different fluorescence signals were detected by real-time fluorescence quantitative PCR.

**Table 1:** Primer probe sequences.

SNP ID	Polymorphism	Probes	Sequences
rs896210	A/G	AA genotype	5′-GCACTTACCATTTGTACGACTTTGA-3′
		GG genotype	5′-GCACTTACCATTTGTACGACTTTGG-3′
		reverse primer	5′-AGGGCTTGAGGGGTATACTGCTTTG-3′
rs1868092	A/G	AA Genotype	5′-TGTATATTCACATAGTGCACTTTGA-3′
		GG genotype	5′-TGTATATTCACATAGTGCACTTTGG-3′
		reverse primer	5′-CCCCTTTCCCTTCGGGACCTCCATC-3′
rs4953396	A/C	AA genotype	5′-CTCTGGGAACTACCCCAGACACCAA-3′
		CC genotype	5′-CTCTGGGAACTACCCCAGACACCAC-3′
		reverse primer	5′-GGTCAGGTGTCTACCTATGGTCCAT-3′
rs4953354	A/G	AA genotype	5′-ACAAGAGCAGGAGAGCCAAGGGGGA-3′
		GG genotype	5′-ACAAGAGCAGGAGAGCCAAGGGGGG-3′
		reverse primer	5′-TTGAAAGTATTTATGGAGCATATCT-3′

### 2.3 Statistical analysis

Statistical analysis was performed using SPSS 25.0 software (*p* < 0.05 statistically different). In the analysis of baseline information, numerical variables were compared between groups using the t-test, described as (x^2^ ± s), and categorical variables were compared using the X^2^ test, expressed as frequencies and percentages. To evaluate the association between EPAS1 and ATP6V1E2 gene polymorphisms and the risk of developing HAPC, this study constructed five genetic models (codominant, dominant, recessive, superdominant, and allele models). Multivariate logistic regression analysis was performed, adjusting for age and sex to control for potential confounding factors. The Hardy–Weinberg equilibrium (HWE) test was used to assess the Population genetics of control group and if *p* > 0.05, it indicated that the data in this study followed the population genetics pattern and was representative of the population.

### 2.4 Other analyses

Linkage disequilibrium determination and haplotype analysis using online SHEsis^1^ to analyze the association between genetic haplotypes and the risk of developing (22). The interactions between the studied loci were analyzed using the multifactor downscaling software MDR 3.0.2 to calculate the cross-validation consistency (CVC) and testing accuracy of each model. A network map of EPAS1 and ATP6V1E2 protein-protein interaction (PPI) was constructed using the STRING website^2^ and Cytoscape software, followed by KEGG pathway analysis using the clusterProfiler package in R software (version 4.4.1) to further explore *EPAS1* and *ATP6V1E2* biological functions. Given the sample size of this study, to assess the reliability of variables that failed to reach statistical significance in the logistic regression analysis, we conducted a post-hoc power analysis. This analysis was performed using the pwr package in R software (version 4.4.1).

## 3 Results

### 3.1 Clinical characteristics of study subjects

The clinical characteristics of these participants are shown in Table 2. The mean age of the control and HAPC patients was 45.76 ± 18.14 and 48.41 ± 14.67 years, respectively, with no significant difference between the groups *(p = 0.306*). The percentage of males in HAPC patients was 76%, which was significantly higher than the percentage of males in the control group (38%), with a significant difference between the groups (*p < 0.001*). In addition, the differences in RBC, HGB and HCT were significantly different between the two groups (*p < 0.001*).

**Table 2:** Analysis of the basic clinical characteristics of the study population.

Characteristic	Control (*n* = 85)	HAPC (*n* = 78)	*p*-value^2^
Gender			<0.001
Male	32 (38%)	59 (76%)	
Female	53 (62%)	19 (24%)	
Age (yaer)	45.76 ± 18.14	48.41 ± 14.67	0.306
RBC (10^9^/L)	4.92 ± 0.57	7.00 ± 0.69	<0.001
HGB (g/L)	143.73 ± 17.31	221.49 ± 18.72	<0.001
HCT (%)	42.48 ± 6.13	64.78 ± 5.83	<0.001

### 3.2 Hardy Weinberg equilibrium (HWE) test analysis

The HWE-P for *ATP6V1E2* rs896210 and *EPAS1* rs1868092, rs4953396, and rs4953354 in both the control and case groups was greater than 0.05, indicating that the gene frequencies observed in this study population were representative of the gene distributions observed in the general population, as shown in Table 3.

**Table 3:** HWE balance of gene loci.

SNPs	Genetic typing	Control			HAPC		
		N	χ^2^	HWE-P	N	χ^2^	HWE-P
rs896210	GG	50	0.084	0.771	38	0.470	0.493
	AG	31			31		
	AA	4			9		
rs1868092	AA	59	0.058	0.809	36	0.117	0.733
	AG	24			33		
	GG	2			9		
rs4953396	AA	48	0.313	0.576	32	0.787	0.357
	AC	33			33		
	CC	4			13		
rs4953354	GG	66	2.303	0.129	34	0.107	0.744
	AG	16			34		
	AA	3			10		

### 3.3 Association analysis of *EPAS1* and *ATP6V1E2* gene locus polymorphisms with HAPC susceptibility

**Figure 2:**
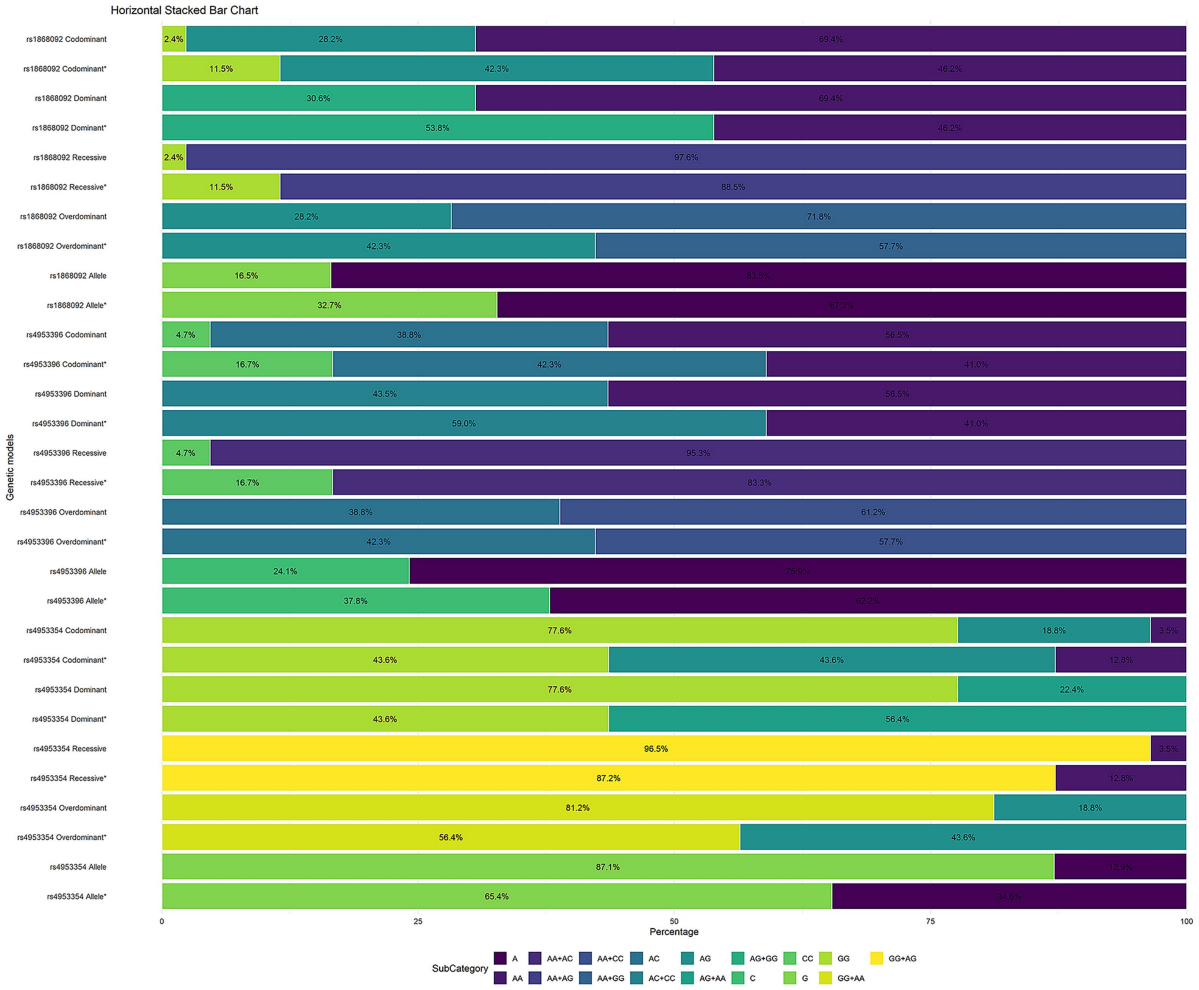
Horizontal stacked bar chart of genetic models; * for HAPC group.

We assessed the association of SNPs in the *ATP6V1E2* and *EPAS1* genes with HAPC by constructing multiple genetic models, including codominant, dominant, recessive, and overdominant models, followed by logistic regression analysis. The final results showed *ATP6V1E2* rs896210 and *EPAS1* rs1868092, rs4953396, rs4953354 were all significantly correlated with HAPC. Genotyping and alleles in the control and HAPC groups are shown in Table 4, and the percentage of genotypes in each gene model is shown in Figure 2.

**Table 4:** Association analysis of gene locus polymorphisms with the risk of developing HAPC.

SNP	Model	Genotype	Control	HAPC	OR (95%CI)	*p* value
rs896210	Codominant	GG	50	38	Reference	
		AG	31	31	1.307 (0.635–2.692)	0.468
		AA	4	9	4.686 (1.160–18.935)	0.030
	Dominant	GG	50	38	Reference	
		AG + AA	35	40	1.609 (0.812–3.189)	0.173
	Recessive	GG + AG	81	69	Reference	
		AA	4	9	4.194 (1.073-16.398)	0.039
	Overdominant	GG + AA	54	47	Reference	
		AG	31	31	1.078 (0.538–2.160)	0.832
	Allele	G	131	107	Reference	
		A	39	49	1.748 (1.032–2.959)	0.038
rs1868092	Codominant	AA	59	36	Reference	
		AG	24	33	2.047 (0.984–4.259)	0.055
		GG	2	9	10.07 (1.765–57.451)	0.009
	Dominant	AA	59	36	Reference	
		AG + GG	26	42	2.551 (1.269–5.129)	0.009
	Recessive	AA+AG	83	69	Reference	
		GG	2	9	7.858 (1.397–44.194)	0.019
	Overdominant	AA+GG	61	45	Reference	
		AG	24	33	1.665 (0.817–3.394)	0.16
	Allele	A	142	105	Reference	
		G	28	51	2.554 (1.429–4.563)	0.002
rs4953396	Codominant	AA	48	32	Reference	
		AC	33	33	1.454 (0.701–3.016)	0.315
		CC	4	13	6.4 (1.689–24.246)	0.006
	Dominant	AA	48	32	Reference	
		AC + CC	37	46	1.924 (0.969–3.822)	0.062
	Recessive	AA+AC	81	65	Reference	
		CC	4	13	5.427 (1.49–19.76)	0.01
	Overdominant	AA+CC	52	45	Reference	
		AC	33	33	1.075 (0.538–2.146)	0.838
	Allele	A	129	97	Reference	
		C	41	59	2.069 (1.261–3.395)	0.004
rs4953354	Codominant	GG	66	34	Reference	
		AG	16	34	9.137 (2.075–40.222)	0.003
		AA	3	10	3.648 (1.652–8.056)	0.001
	Dominant	GG	66	34	Reference	
		AG + AA	19	44	4.38 (2.085–9.197)	<0.001
	Recessive	GG + AG	82	68	Reference	
		AA	3	10	2.852 (1.327–6.13)	0.007
	Overdominant	GG + AA	69	44	Reference	
		AG	16	34	6.136 (1.418–26.55)	0.015
	Allele	G	148	102	Reference	
		A	22	54	3.806 (2.003–7.231)	<0.001

The distribution of the *ATP6V1E2* rs896210 genotype is as follows: in the control group, *GG* accounted for 58.8%, *AG* for 36.5%, and *AA* for 4.7%; whereas in the HAPC group, *GG* accounted for 48.7%, *AG* for 39.7%, and *AA* for 11.5%. Compared with allele *G*, allele *A* [OR = 1.748 (1.032–2.959), *p* = 0.038] was associated with an increased risk of HAPC. In the codominant model, the *AA* genotype [OR = 4.686 (1.160–18.935), *p* = 0.030] significantly increased HAPC risk compared to the *GG* genotype. In the recessive model, individuals carrying the *AA* genotype [OR = 4.194 (1.073–16.398), *p* = 0.039] also exhibited increased susceptibility to HAPC.

The genotype distribution of *EPAS1* rs1868092 was as follows: in the control group, 69.4% *AA*, 28.2% *AG*, and 2.4% *GG*, whereas in the HAPC group, 46.2% *AA*, 42.3% *AG*, and 11.5% *GG*. Using allele *A* as a reference, allele *G* [OR = 2.554 (1.429–4.564), *p* = 0.002] was associated with the risk of developing HAPC. In a codominant model with the *AA* genotype as reference, the *GG* genotype increases the risk of HAPC [OR = 10.070 (1.765–57.451), *p* = 0.009]. Individuals carrying the *G* genotype (*AG+GG*) [OR = 2.551 (1.269–5.129), *p* = 0.009] in the dominant model and the *GG* [OR = 7.858 (1.397–44.194), *p* = 0.019] genotype in the recessive model were both more likely to develop HAPC.

The genotypes of *EPAS1* rs4953396 were distributed as follows: in the control group, 56.5% were *AA*, 38.8% were *AC*, and 4.7% were *CC*, whereas in the HAPC group, 41.0% were *AA*, 42.3% were *AG*, and 16.7% were *GG*. Using allele *A* as a reference, allele *C* [OR = 2.069 (1.261–3.395), *p* = 0.004] was associated with an increased risk of developing HAPC. The *CC* [OR = 6.400 (1.689–24.246), *p* = 0.006] genotype in the codominant model could be significantly associated with an increased risk of HAPC compared to the *AA* genotype. Individuals carrying the *CC* [OR = 5.427 (1.490–19.760), *p* = 0.010] genotype in the recessive model were both more likely to develop HAPC.

The genotype distribution of *EPAS1* rs4953354 was as follows: in the control group, *GG* accounted for 77.6%, *AG* for 18.8%, and *AA* for 3.5%, whereas *GG* accounted for 43.6%, *AG* for 43.6%, and *AA* for 12.8% in the HAPC group. The allele *A* [OR = 3.806 (2.003–7.231), *p* < 0.001]for this SNP, *AG* [OR = 9.137 (2.075–40.222) and *AA* [OR = 3.648 (1.652–8.056), *p* = 0.001] genotypes in the codominant model, and *AG+AA* in the dominant model [OR = 4.380 (2.085–9.197), *p* < 0.001]genotype, *AA* [OR = 2.852 (1.327–6.130), *p* = 0.007]genotype in the recessive model, and *AG* [OR = 6.136 (1.418–26.550), *p* = 0.015] genotype in the overdominant model significantly increased HAPC susceptibility.

### 3.4 Haplotype and linkage disequilibrium analysis of *EPAS1* and *ATP6V1E2* gene SNPs with HAPC

**Figure 3:**
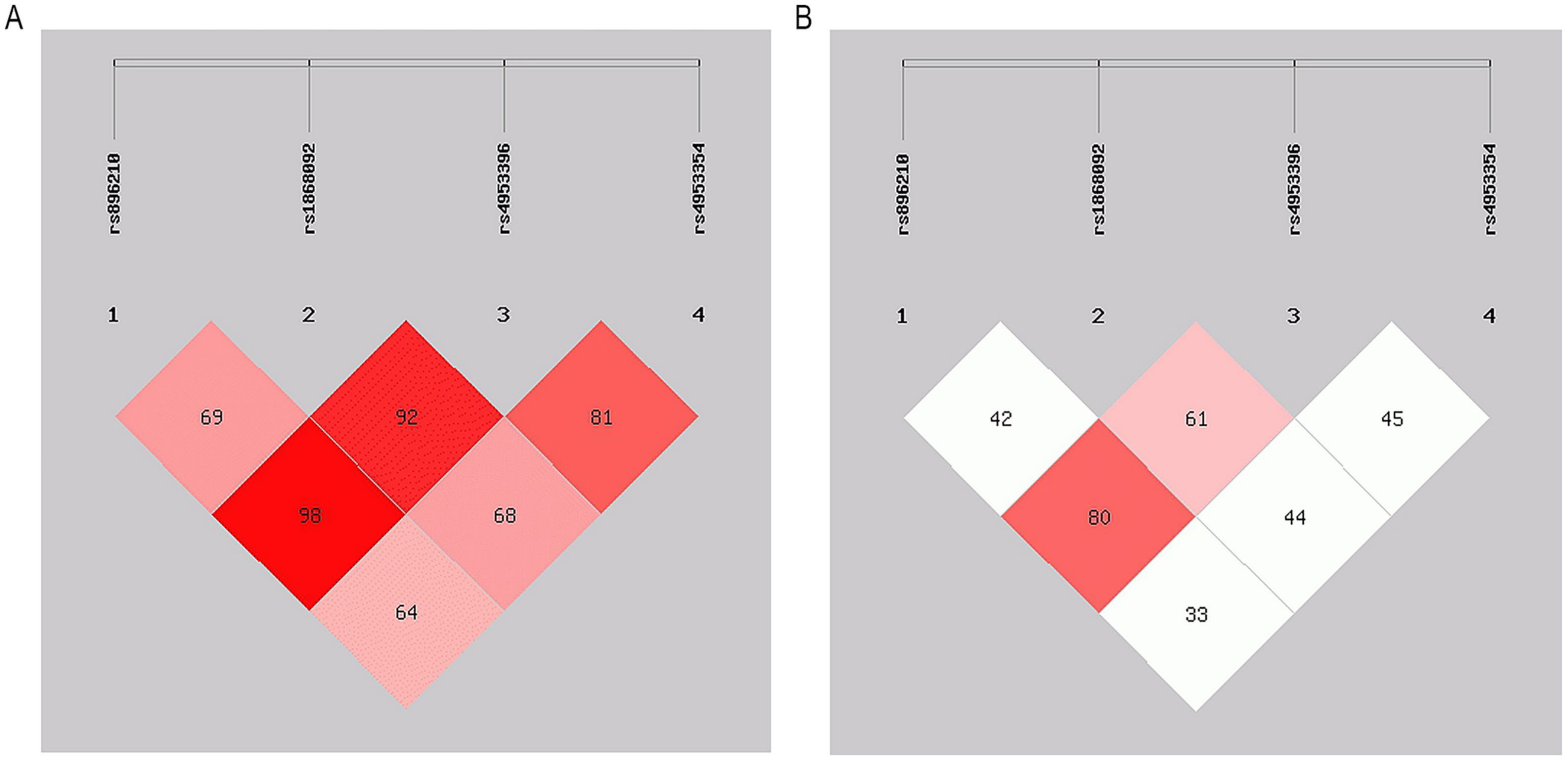
Linkage disequilibrium (LD) of 5 SNPs. **(A)** The numbers inside the diamonds indicate the D’ for pairwise analyses. **(B)** The numbers inside the diamonds indicate the r^2^ for pairwise analyses.

We performed haplotype analysis of these four SNPs using online SHEsis, with the SNPs in the order of rs896210, rs1868092, rs4953396, and rs4953354. After removing the haplotypes with frequencies lower than 3%, a total of six haplotypes were obtained, *GAAG*, *AGCA*, *GGCA*, *GAAA*, *AACG*, and *AGCG* (in descending order of frequency in the HAPC group), as shown in Table 5. Among them, *GAAG* (OR = 0.437 [0.268–0.715], *p < 0.001*), *AGCA* (OR = 2.871 [1.485–5.552], *p = 0.001*), and *GGCA* (OR = 9.973 [1.713–58.064], *p = 0.012*) differed significantly between the HAPC and control groups. *GAAG* appeared significantly less frequently in the HAPC group than in the control group, so the risk of HAPC was significantly lower in *GAAG* haplotype carriers, whereas the risk of HAPC would be significantly higher in *AGCA* and *GGCA* haplotype carriers. In addition, a high degree of linkage disequilibrium between rs896210 and rs4953396 can be seen in Figure 3 (D′ = 0.98, r^2^ = 0.80).

**Table 5:** Haplotype analysis of gene locus polymorphisms and risk of HAPC occurrence.

Haplotypes	HAPC group	Control group	χ^2^	*p* value	OR (95%CI)
GAAG	87.28 (55.9%)	124.58 (73.3%)	11.074	<0.001	0.437 [0.268 ~ 0.715]
AGCA	33.06 (21.2%)	14.85 (8.7%)	10.414	0.001	2.871 [1.485 ~ 5.552]
GGCA	8.33 (5.3%)	0.97 (0.6%)	6.202	0.012	9.973 [1.713 ~ 58.064]
GAAA	6.51 (4.2%)	2.30 (1.4%)	2.515	0.113	3.216 [0.703 ~ 14.715]
AACG	6.17 (4.0%)	10.22 (6.0%)	0.687	0.407	0.650 [0.233 ~ 1.812]
AGCG	5.77 (3.7%)	9.03 (5.3%)	0.463	0.496	0.691 [0.237 ~ 2.014]

### 3.5 SNP-SNP interaction analysis

**Figure 4:**
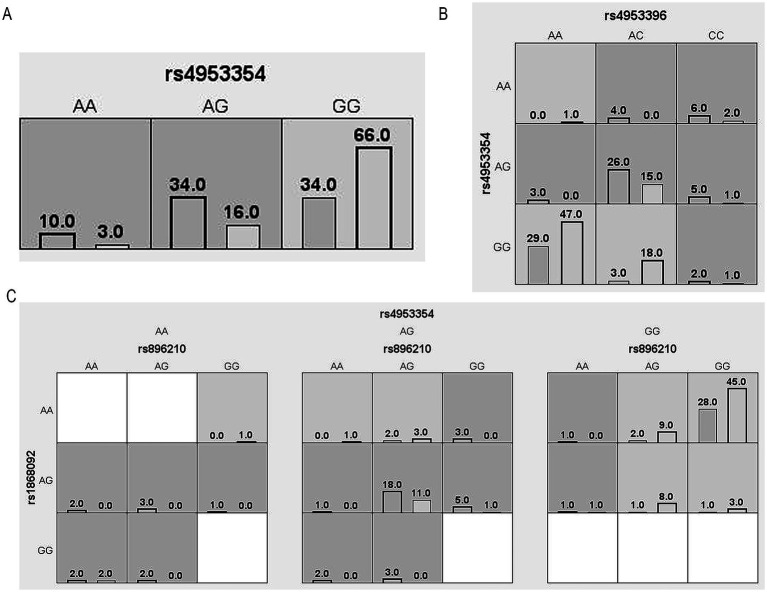
Cell diagram of the optimal model. **(A)** rs4953354 model; **(B)** rs4953396, rs4953354 model; **(C)** rs1868092, rs4953396, rs4953354 model. Black bars on the left side indicate the case group, black bars on the right side indicate the control group, one cell represents one interaction combination, light gray cells indicate that the ratio of the combination does not exceed the ratio threshold and is low-risk, dark gray indicates that the ratio threshold is exceeded and is high-risk, and white indicates that there is no data on the combination.

**Figure 5:**
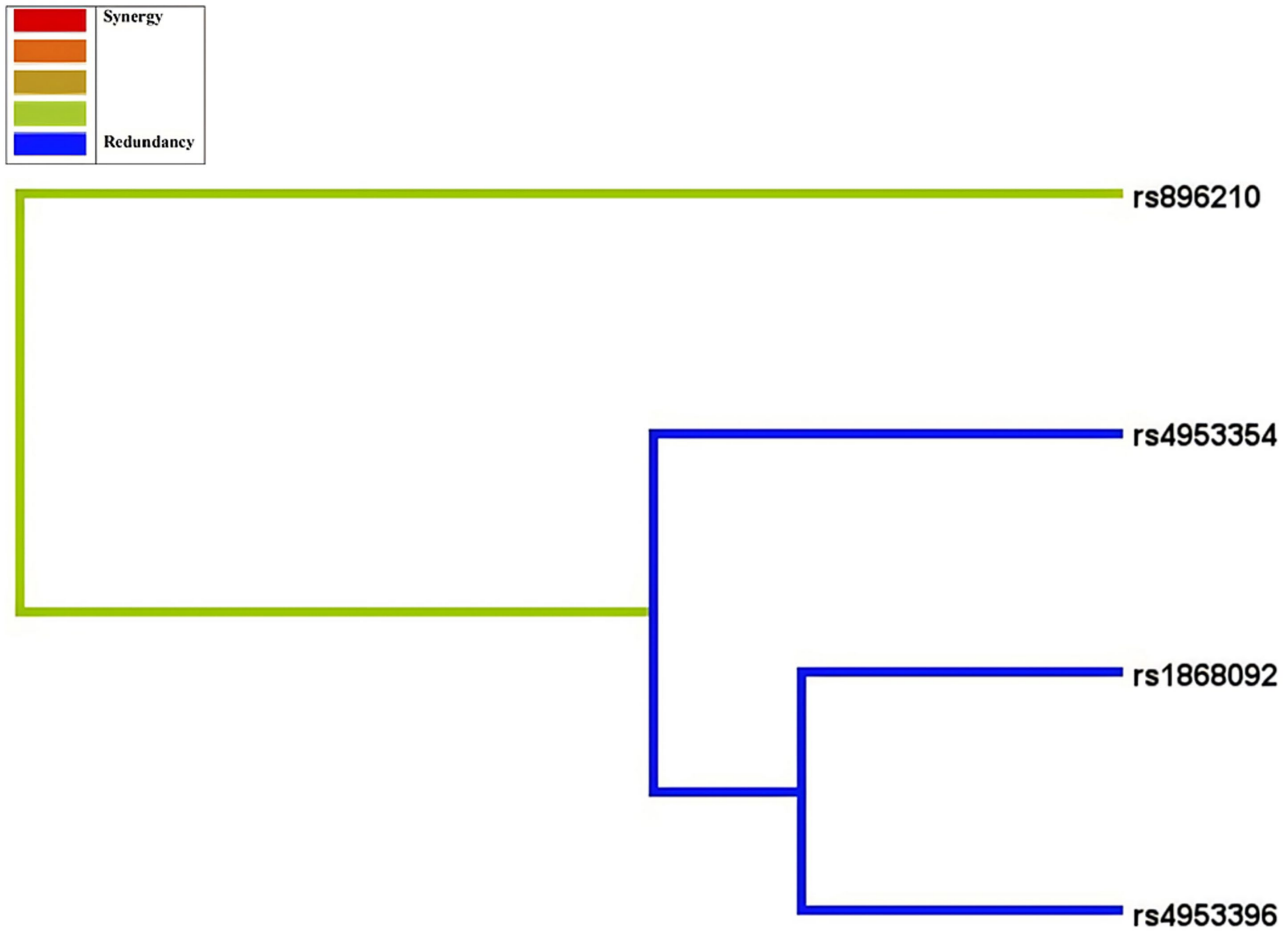
Dendrogram of SNP-SNP interactions. The blue line indicates that the SNPs have a redundancy effect in regulating the risk of HAPC and the green line represents the intermediate point between synergistic and redundancy effects. The closer the loci are, the stronger the interactions are.

We performed MDR analysis on these four SNPs, to explore the interactions between these loci and their associations with HAPC, and the optimal models obtained from 1st-3rd order interactions are shown in Figure 4 and Table 6. The association between the interactions among these SNPs and the risk of HAPC prevalence is statistically significant (*p* < 0.01). The rs4953354 model (testing accuracy: 0.6703, OR = 4.4954, CVC: 10/10) showed that risk allele A at the rs4953354 locus elevates the risk of developing HAPC up to 4.4954-fold. The rs4953396, rs4953354 model (testing accuracy: 0.6772, OR = 4.9934, CVC: 9/10) suggests that when an individual carries the risk allele for both loci, the risk of developing HAPC is 4.9934 times higher than that of an individual who does not carry the allele. The rs896210, rs1868092, rs4953354 model (testing accuracy: 0.6329, OR = 6.032, CVC: 7/10) had moderate test accuracy, there was a high stability (CVC = 7/10) and a strong effect size, which still accounted for the risk of developing HAPC when the risk alleles at the 3 loci coexistedis elevated by a factor of 6.0392. The results showed that the interaction of these SNPs significantly increased the risk of developing HAPC, with rs4953354 being the core risk locus. Figure 5 shows the obtained dendrogram, which shows that redundant effects of rs4953354, rs1868092, and rs4953396 in regulating HAPC risk, and both synergistic and redundant effects between rs896210 and these three loci. And rs896210 was more strongly associated with rs4953354 than the other two loci.

**Table 6:** Multi-factor dimensionality reduction analysis.

Model	Training accuracy	Testing accuracy	*p* value	OR (95%Cl)	CVC
rs4953354	0.6703	0.6703	<0.01	4.4954 (2.2803,8.8621)	10/10
rs4953396, rs4953354	0.6831	0.6772	<0.01	4.9934 (2.5269,9.8677)	9/10
rs896210, rs1868092, rs4953354	0.6963	0.6329	<0.01	6.0392 (2.9542,12.3458)	7/10

### 3.6 Functional association of *EPAS1* and *ATP6V1E2* in HAPC

**Figure 6:**
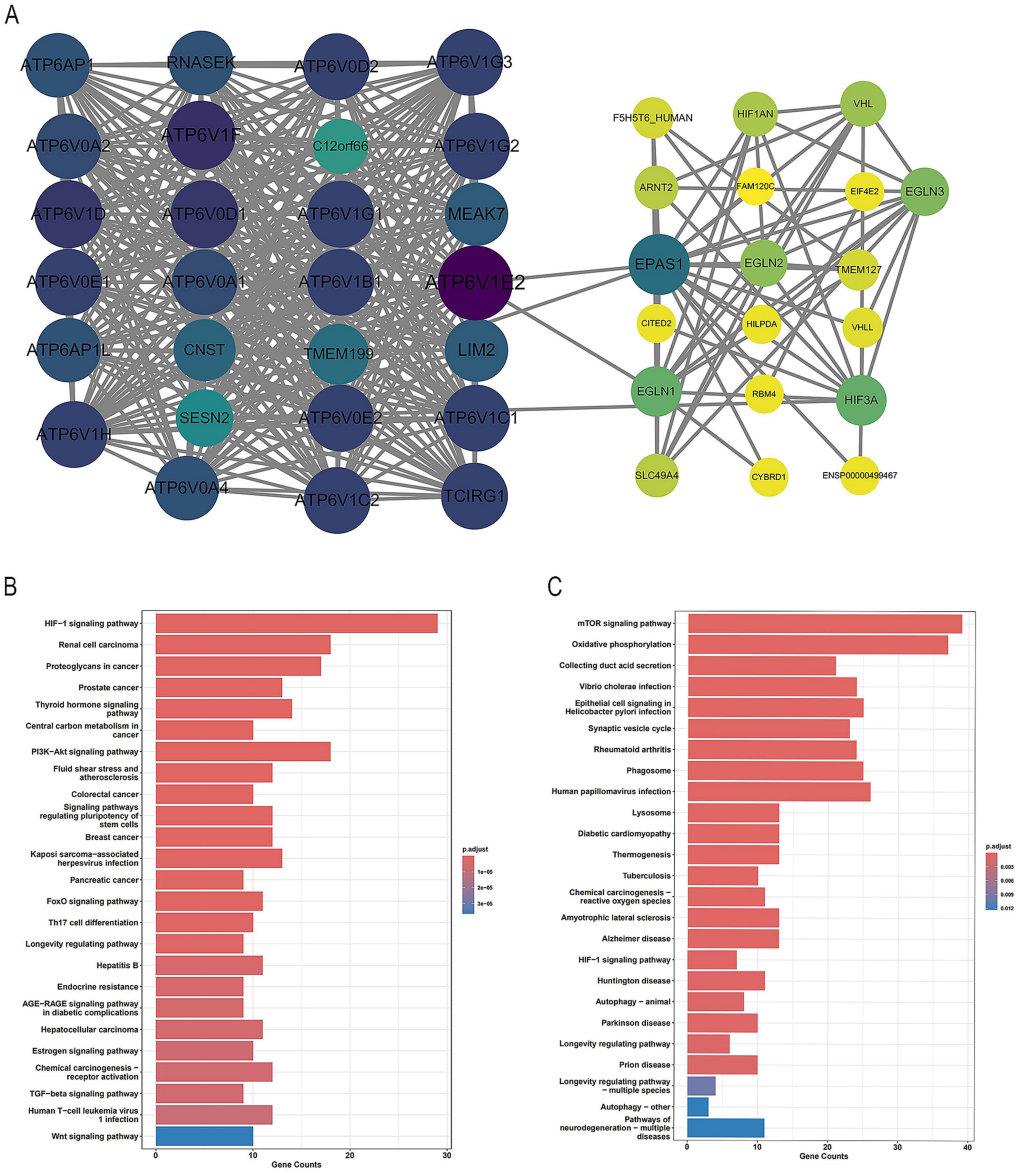
Biological functions of *EPAS1* and *ATP6V1E2*. **(A)** Protein interaction map of *EPAS1*. **(B)** KEGG passage results of proteins related to *EPAS1*. **(C)** KEGG passage results of proteins related to *ATP6V1E2*.

To further explore the potential mechanisms of *EPAS1* and *ATP6V1E2* in regulating HAPC, we first constructed *EPAS1*-*ATP6V1E2* PPI to analyze the direct and indirect molecular associations between the two, and then revealed the biological pathways in which they are jointly involved by KEGG pathway analysis, and Figure 6 shows the final results obtained. The PPI plot (Figure 6A) shows that there is an interaction between *EPAS1* and *ATP6V1E2*. They are both directly linked and indirectly functionally associated through some intermediary molecules (e.g., EGLN1, HIF3α). KEGG pathway analysis (Figures 6A,B) also showed that both *EPAS1* and *ATP6V1E2* and their related proteins were involved in the HIF-1 signaling pathway, in addition to *EPAS1* being involved in signaling pathways related to tumors such as renal, prostate and colorectal cancers, whereas *ATP6V1E2* was involved in the mTOR signaling pathway, oxidative phosphorylation, and collector tubular acid secretion pathways.

## 4 Discussion

At present, HAPC remains a serious threat to the health of highlanders. In China, its main treatments include phlebotomy and therapeutic erythrocyte dialysis, and there is still a lack of effective means of curing the disease (23). Several studies have shown that Chinese Tibetans have a lower incidence of HAPC compared to other highlanders and lowlanders living at the same altitude, a phenomenon closely related to genetic factors (8, 17, 24). Therefore, an in-depth study of HAPC from a genetic perspective is important for the prediction, diagnosis, treatment, and prevention of this disease.

Low-pressure hypoxia due to altitude is the main cause of HAPC. Although the pathogenesis of HAPC remains unclear, the widely accepted hypothesis is that HIF-1 upregulates EPO secretion during exposure to hypoxia at high altitude, leading to increased erythrocyte (25). HIF-1 belongs to the PAS family of hypoxia-regulated transcription factors and consists of an oxygen-sensitive *α*-subunit (HIF-α) and a constitutively expressed *β*-subunit (HIF-β, also known as ARNT) (26). Under normoxic conditions, prolyl hydroxylases (PHDs) hydroxylate key proline residues of HIF-1α and HIF-2α using oxygen molecules and α-ketoglutarate as substrates (27). Hydroxylated HIF-α is recognized by von Hippel–Lindau to form the E3 ubiquitin ligase complex, which is then degraded via the proteasome pathway (28, 29). In contrast, under hypoxic conditions, this oxygen-dependent pathway ceases, resulting in the intracellular stabilization and accumulation of HIF-1α and HIF-2α and the formation of a heterodimer with ARNT, which then binds to the hypoxia-responsive element, locating within the regulatory element of the HIF target gene (30, 31). HRE activates the expression of hypoxia-related genes, such as target genes of EPO, vascular endothelial growth factor, and lactate dehydrogenase A (18). In particular, upregulation of EPO production promotes increased erythropoiesis, a molecular mechanism that links tissue hypoxia to a compensatory erythropoietic response. It has been found that treatment of HAPC rats with the HIF-2α inhibitor PT2385 resulted in significant reductions in the levels of EPO, HGB, RBC, and HCT compared with the untreated group (32). This result suggests that PT2385 may directly regulate red lineage hematopoiesis by inhibiting the HIF-2α-EPO pathway, thereby reducing the abnormal elevation of HGB and RBCs (32).

Oxygen-dependent regulation of HIF-2α is a central mechanism for cellular adaptation to chronic hypoxia, and it plays a key role in the transcriptional regulation of EPO (33). HIF-2α is encoded by *EPAS1* gene, whose function is critical for hypoxic adaptation. Our functional studies of *EPAS1* gene also indicate that *EPAS1*, as a core transcriptional regulator of the hypoxic response, plays a key role in hypoxic adaptation by interacting with proteins such as ARNT, HIF-1α, and TP53, and by integrating regulatory mechanisms such as hypoxic signaling (HIF-1) and other mediators. Specific mutations in *EPAS1* (e.g., Tibetan-adapted mutations) reduce the incidence of HAPC, whereas certain mutations may increase HAPC susceptibility. Using population genetic analysis, we found that polymorphisms at *EPAS1* rs1868092, rs4953396 and rs4953354 were significantly associated with HAPC in the Chinese Tibetan population. Among them, rs1868092-G, rs4953396-C and rs4953354-A were risk alleles for HAPC, and genetic modeling analysis showed that the mutant genotypes of these three SNPs significantly increased the susceptibility to HAPC in codominant, dominant and recessive models. In addition, the rs4953354 locus showed a super dominant effect. This finding is consistent with previous conclusions, both of which indicate that there is a significant correlation between *EPAS1* gene polymorphisms and susceptibility to HAPC (34, 35).

This study identified a significant association between the ATP6V1E2 rs896210 polymorphism and the risk of HAPC. This gene encodes a key subunit of the V-ATPase, which is essential for maintaining lysosomal acidification. An unexpected study on Hif-2a conditional knockout mice provided key insights into its function: in this mouse model, downregulation of ATP6V1E2 expression directly led to impaired lysosomal acidification (36). This suggests that one potential mechanism of ATP6V1E2 in high-altitude adaptation may be: its variants influence lysosomal acidification and function (e.g., protein degradation, autophagy) by regulating V-ATPase activity, thereby indirectly modulating the stability and turnover of hypoxia-responsive proteins like HIF-*α*. Ultimately, this forms a synergistic regulatory network with EPAS1. Concurrently, this study confirmed through KEGG pathway analysis that both EPAS1 and ATP6V1E2 are enriched in the HIF-1 signaling pathway, indicating that they synergistically participate in the development of HAPC via this critical hypoxia response pathway. Although direct functional studies of ATP6V1E2 remain limited, its significance has been validated through multiple lines of evidence: not only is it located adjacent to the EPAS1 gene and shares a selective signal (9), but this study further directly confirmed their presence within a tightly integrated functional network through PPI network analysis. Collectively, these findings establish ATP6V1E2 as a key player in the hypoxic adaptation regulatory network.

This study also revealed the synergistic contribution of *ATP6V1E2* and *EPAS1* loci to Tibetan high-altitude adaptation through haplotype and linkage disequilibrium analysis. We identified two functionally distinct haplotypes: *AGCA* and *GGCA* as risk haplotypes for HAPC, while *GAAG* exhibits protective effects. Further linkage analysis confirmed strong linkage disequilibrium between rs896210 and rs4953396. Crucially, multiple interaction model analysis demonstrated robust statistical interactions among the four SNP loci, collectively determining HAPC risk. This suggests polygenic interactions are more critical than single-gene variants. This finding aligns with genomic evidence implicating polygenic regions (Genes such as *ATP6V1E2*, *TMEM247*, *RHOQ*, *PIGF*, and *CRIPT*) in high-altitude adaptation (9, 37), offering new insights into the functional role of this gene cluster. Consequently, future research may explore genetic testing for screening high-risk populations and investigate potential drug targets based on these genetic variations, providing novel strategies for HAPC prevention and precision treatment.

However, this study has several limitations. First, the subjects were exclusively Tibetan individuals from the Lhasa region of Tibet, potentially limiting the generalizability of the findings. Future studies should include populations from different geographic regions and ethnic groups to validate broader applicability. Second, the limited sample size, but post-hoc power analysis indicates sufficient detection capability for strong genetic effects (OR > 4.0), with some loci exhibiting >90% power, ensuring the reliability of major positive findings. However, the power for detecting weak effects (OR < 1.5) was low (most < 20%), requiring cautious interpretation of negative results. Future studies should expand sample sizes to enhance statistical power for detecting subtle genetic effects. Finally, this study focused solely on limited sites within the ATP6V1E2 and EPAS1 genes, which to some extent limited the comprehensive assessment of polygenic interactions. Future research should conduct deep saturation mutation screening of these two genes and subsequently extend the analysis to other members of the EPAS1 gene cluster to elucidate the polygenic inheritance architecture of HAPC at the systems level.

## 5 Conclusion

This study found that specific SNPs at the *ATP6V1E2* and *EPAS1* loci are not only independently associated with HAPC susceptibility but also exhibit significant genetic interactions, thereby providing new evidence for the polygenic interaction hypothesis. More importantly, establishing risk assessment models based on these SNPs holds promise for providing a theoretical basis for early screening and personalized prevention and treatment of HAPC among the Tibetan population in this region.

## Data Availability

The original contributions presented in the study are included in the article, further inquiries can be directed to the corresponding authors.

## Footnotes

1
http://analysis.bio-x.cn/myAnalysis.php

2
https://cn.string-db.org/
